# Effects of Oral Administration of Goat Rumen-Derived Bacterial Isolates on In Vitro and In Vivo Rumen Fermentation Characteristics

**DOI:** 10.3390/ani16132091

**Published:** 2026-07-06

**Authors:** Yushu Zhang, Yutaka Uyeno

**Affiliations:** Graduate School of Science and Technology, Shinshu University, Minamiminowa, Nagano 399-4511, Japan; 23hs552b@shinshu-u.ac.jp

**Keywords:** community analysis, in vitro fermentation, lactic acid producing bacteria, rumen microbial community

## Abstract

Rumen-derived isolates are promising direct-fed microbial candidates for modulating rumen fermentation in ruminants. However, the effects of goat rumen-derived isolates have not been well characterized. In this study, two experiments were conducted to evaluate the effects of supplementation with two rumen-derived isolates identified as *Pediococcus* sp. (PE) and *Streptococcus* sp. (ST) on goat rumen fermentation. In Experiment 1, each isolate was tested separately against a non-supplemented control at three levels of inoculation (10^4^, 10^6^, and 10^8^ CFU/mL) in a 24 h in vitro rumen fermentation assay. In Experiment 2, a 3 × 3 Latin square feeding trial was conducted to evaluate the effects of long-term supplementation with the selected isolates on the rumen fermentation characteristics in goats. The feeding trial consisted of three 21 d experimental periods separated by 5 d washout intervals. Goats were fed without an additive (Control) or supplemented with 1 mL of PE (10^7^ CFU/mL) or ST (10^9^ CFU/mL) at 1 mL/day. In Experiment 1, both isolates increased the proportion of propionate (PA%) and decreased the acetate-to-propionate ratio (A/P). PE significantly increased total gas and methane production. The high dose of ST significantly decreased the relative abundances of Prevotellaceae and *Xylanibacter* but increased those of Fibrobacteraceae and *Fibrobacter*. In Experiment 2, isolate-associated changes in microbial co-occurrence networks were evaluated, resulting in the alteration of the dominant microbial modules. Results suggest that the isolates may have influenced rumen fermentation by reshaping potential interaction patterns among rumen microorganisms. These findings indicate that rumen-derived isolates have potential as modulators of rumen fermentation in goats, and the responses to supplementation are influenced by isolate type and the supplementation level.

## Simple Summary

This study evaluated the effects of two rumen-derived lactic acid bacteria isolates, *Pediococcus* sp. and *Streptococcus* sp., on goat rumen fermentation through in vitro and long-term feeding trials. In vitro, both isolates increased propionate proportion (PA%) and decreased the acetate-to-propionate ratio. Long-term in vivo supplementation improved basal fermentation status, increasing ammonia nitrogen concentrations. Ex vivo fermentation further confirmed that both isolates significantly increased PA% and reduced archaeal abundance. Co-occurrence network analysis revealed that continuous supplementation reshaped potential interaction patterns among rumen microorganisms, shifting the fermentation pathway of substrate digestion rather than merely altering specific taxa abundance. Both isolates demonstrated potential as modulators of rumen fermentation in goats. The responses to supplementation were highly influenced by the specific type of isolate and the dosage level. These findings underscore the importance of utilizing microbial additives in ruminants to optimize gastrointestinal fermentation and enhance production efficiency.

## 1. Introduction

Rumen fermentation degrades fiber and other complex carbohydrates into short-chain fatty acids via the microbial community, providing energy and nutrients to the host [1,2]. Rumen fermentation and microbiota modulation can influence host metabolism [3]. Direct-fed microbials (DFMs) and probiotics have been widely investigated in ruminants and are considered promising approaches toward improving feed utilization and fermentation efficiency [4], while also promoting the overall health and stabilization of gastrointestinal microbiota, sometimes as an alternative to antibiotics [5]. In particular, supplementation with lactic acid bacteria (LAB) improves the nutritional status of ruminants by increasing the digestibility of nutrients, promoting volatile fatty acid (VFA) production, and providing more nitrogen sources for microbial protein synthesis [6]; *Pediococcus* and *Streptococcus* are widely recognized LAB known to affect intestinal fermentation [7,8].

In vitro studies have suggested that *Pediococcus* spp. and *Pediococcus*-containing probiotics can modulate rumen fermentation and show methane-reducing potential, although their effects depend on strains and substrates [9,10,11]. Feeding studies further reported that fermented feed containing *Pediococcus* increased milk yield in dairy cows, while a commercial compound probiotic including this genus altered calf rumen fermentation and development, together with improved calf health [12,13,14]. Some studies have suggested that *Streptococcus* may also be involved in fiber degradation, because some members of this genus showed xylanase and CMCase activity [15] and possess cellulolytic-related enzyme system with the capacity to degrade polysaccharides derived from the plant cell wall [16]. Rumen-derived *Streptococcus* strains or probiotic-containing *Streptococcus* strains have also been suggested to have potential to alter rumen physiological parameters and to improve nutrient digestibility as well as production level in ruminants [17,18,19].

The application of DFMs in ruminants includes inconsistent responses, unclear long-term effects, and limited in vivo viability; therefore, the specific selection of host-adapted strains and further investigation of their interactions with the host resident microbiota are required [20]. Based on this rationale, we previously isolated a *Pediococcus* sp. strain (PE) and *Streptococcus* sp. strain (ST) from the goat rumen, which showed promise as direct-fed bacteria exerting different fermentation-modulating potential [21]. We hypothesized that supplementation with these two rumen-derived isolates would improve rumen fermentation characteristics and modulate microbial communities, and these effects would remain after continuous feeding. The objective of this study was to evaluate these two rumen-derived isolates on fermentation characteristics and microbial communities across different systems (Experiment 1, in vitro evaluation; Experiment 2, feeding trial), thereby providing new information for the screening and functional evaluation of candidate DFMs in ruminants and revealing their potential mechanisms.

## 2. Materials and Methods

### 2.1. Experiment 1—In Vitro Evaluation

#### 2.1.1. Isolate Preparation

The strains used in this study were originally isolated from the rumens of adult goats, including PE (isolate B) and ST (isolate 14). These isolates were selected based on their effects on preliminary in vitro rumen fermentation [21]. Based on 16S rRNA gene sequencing and BLAST web tool (National Center for Biotechnology Information, Bethesda, MD, USA; https://blast.ncbi.nlm.nih.gov/Blast.cgi accessed on 15 April 2026) analysis, isolate B and isolate 14 were conservatively assigned as *Pediococcus* sp. and *Streptococcus* sp., respectively. The closest BLAST matches and conservative taxonomic assignments are provided elsewhere [21]. For both in vitro and in vivo experiments, the isolates were grown in de Man, Rogosa, and Sharpe (MRS; Oxoid, Basingstoke, UK) for PE and Gifu Anaerobic Medium (GAM; Nissui Pharmaceutical Co., Ltd., Tokyo, Japan) for ST broth and at 39 °C for 24 h under anaerobic conditions. Living cells of the isolates were harvested by centrifugation at 5000× *g* for 10 min at 4 °C, and resuspended in sterile saline for use in the in vitro experiment and feeding trial. Concentration was measured based on the number of colony-forming units (CFUs)/mL on a plate counter. Before use, the isolates were stored at 4 °C.

#### 2.1.2. In Vitro Fermentation Experiment

Animal handling was performed according to the Shinshu University guidelines (Approval no. 23120). Rumen liquid was obtained from three adult Japanese Shiba goats (three dry and non-pregnant females (average BW, 38.8 kg) for PE experiment; two dry and non-pregnant females and one castrated male (average BW, 48.3 kg) for ST experiment) through a stomach tube. Goats were fed oat hay and alfalfa hay cubes at 1:1 ratio twice daily, and the feed amount was adjusted according to the body weight of each goat, with free access to water. The rumen fluids were collected before morning feeding and stored in a thermos bottle at 39 °C. To avoid saliva contamination, the first 50 mL of the rumen fluid was discarded. The rumen fluid was squeezed through four layers of gauze, mixed with buffer at a 1:1 ratio, and flushed with CO_2_ gas. The fermentation substrate consisted of oat hay and alfalfa hay cubes at 1:1 ratio, corresponding to the daily feed. Ground substrate samples (0.35 g of DM) were incubated in 100 mL bottles with 35 mL of mixed rumen buffer fluid. The PE and ST in vitro fermentation experiments were conducted separately on different days as two independent experiments. Within each in vitro experiment, the experimental groups included a non-supplemented control and three inoculation levels of the corresponding isolate, and the datasets were analyzed separately. For each donor animal, two replicate bottles were prepared for each treatment. The isolates were supplemented at three levels (10^4^, 10^6^, and 10^8^ CFU/mL). After dispensing, bottles were closed with rubber stoppers, O_2_ was removed by flushing with N_2_ gas, and the samples were then shaken automatically at 75 rpm and incubated at 39 °C for 24 h. The total gas production assay was conducted as described previously [22]; at 6 and 24 h, 5 mL of gas was obtained from each bottle and infused into a vacuum tube to measure the concentration of CH_4_. After incubation, the liquid was collected for measuring pH, NH_3_-N, and VFAs. Samples were also collected to quantify total bacteria and methanogenic archaea by quantitative PCR. In addition, selected samples were subjected to 16S rRNA gene sequencing for microbial community analysis.

### 2.2. Experiment 2—Feeding Trial

#### 2.2.1. Experimental Design

The in vivo experiment was also carried out at the Shinshu University farm. Three young Japanese Shiba goats (one male and two females), aged approximately 18 months with an initial body weight of 16.0 ± 2.4 kg, were assigned randomly to a Latin square design feeding trial for 78 days. The goats were housed in pens equipped with feeders and automatic drinkers and had free access to fresh water throughout the experiment. All animals were offered a diet based on forage containing oats, hay, and an alfalfa hay cube at a 1:1 ratio, meeting the requirements for their standard growth in addition to their maintenance level based on the BW of each goat. All food refused at each feeding was collected.

A 3 × 3 Latin square design was employed, with three supplementary treatments, three periods, and three goats per square. Each experimental period lasted 21 days; the last 4 days were dedicated to data and sample collection, followed by a 5-day washout interval before the next period.

The three treatments were as follows: (1) Control (without isolate supplementation), (2) ST (1.79 × 10^9^ CFU/mL), and (3) PE (2.84 × 10^7^ CFU/mL). Each strain was supplied with isolate at 1 mL/day in a commercial capsule made by plant-origin carbohydrates and offered orally via the morning feeding. The isolate doses were determined based on the results obtained from Experiment 1. Diets were offered twice daily at 09:00 and 15:00 h.

#### 2.2.2. Sampling in Feeding Trials

The amounts of feed offered and refused were weighed and recorded weekly to calculate dry matter intake (DMI). Goats were weighed at the beginning and end of each period before the morning feeding. The average daily gain (ADG) and feed conversion ratio (FCR; DMI/ADG) were then calculated for each period. Fecal samples were also collected over the final four days of each period to measure fecal VFAs, NH_3_-N, and the microbial community. On the final day of each experimental period, rumen fluid samples were collected after morning feeding using an oral catheter. The collected rumen fluid was immediately filtered through four layers of gauze flushed with CO_2_. The in vitro cultivation was conducted immediately using the adapted rumen fluid mixed with McDougall’s buffer [23] at a ratio of 1:1. The fermentation was performed separately for each goat’s rumen liquid, using the same substrate and incubation conditions as those described for Experiment 1. Filtered rumen fluid was mixed with pre-warmed buffer under anaerobic conditions and transferred into sealed fermentation bottles containing 0.35 g of the same substrates without the inoculum. The bottles were incubated at 39 °C for 24 h with shaking. The liquor collected after 24 h of incubation was used in Experiment 1.

The original rumen fluid was stored at −20 °C until analyses of pH, VFAs, NH_3_-N, qPCR, and 16S rRNA gene amplicon sequencing were conducted.

### 2.3. Sample Analysis and Measurements

The pH of the rumen fluid and fermentation culture was measured immediately after sample collection using a digital pH meter (LAQUAtwin-pH-22, HORIBA, Kyoto, Japan). VFAs were analyzed using an LC-2000 system (JASCO Corporation, Tokyo, Japan) under the conditions described in our previous study [22], and NH_3_-N was measured using a commercial kit (F-Kit Ammonia, Roche Diagnostics, Tokyo, Japan) as described previously [24]. In vitro digestibility was determined after 24 h of incubation using the residue of filtration through pre-weighed quantitative filter paper (grade 5A, 110 mm in diameter; ADVANTEC, Toyo Roshi Kaisha, Ltd., Tokyo, Japan), followed by drying at 65 °C for 48 h, and calculated as follows:

IVDMD (%) = [1 − ((DM residue sample − DM residue blank)/initial DM of the substrate)] × 100

For fecal analysis, 2 g of each fecal sample was diluted with 10 mL of distilled water and thoroughly homogenized. After standing for 3 h at 4 °C, the fecal suspension was sampled to allow for the determination of pH, VFA, NH_3_-N, and amounts of bacteria and archaea. The VFA, NH_3_-N, DNA extraction, and qPCR analysis procedures were the same as those used for the rumen fluid.

The total abundances of bacteria and archaea were quantified by real-time PCR. DNA extraction was performed for microbial analysis using the QIAamp DNA Stool Mini Kit (Qiagen, Hilden, Germany), following the manufacturer’s instructions, and stored at −20 °C until analysis. The primer sets Eub338F (ACTCCTACGGGAGGCAG) and Eub522R (ACGTCRTCCMCNCCTTCCTC) were used to quantify total bacteria, whereas qmcrA-F (TTCGGTGGATCDCARAGRGC) and qmcrA-R (GBARGTCGWAWCCGTAGAATCC) were used for archaea by quantifying a gene involved in methane production (mcrA) using the CFX96™ real-time system (Bio-Rad Inc., Hercules, CA, USA) and the SYBR(R) Premix Ex Taq™ Kit (Takara Bio Inc., Otsu, Japan). In total, 40 cycles were performed, with each cycle including denaturation at 95 °C for 10 s, annealing at 60 °C for 20 s, and extension at 72 °C for 30 s for 35 cycles. Dissociation and melting curve analyses were performed to confirm the expected PCR end products.

The extracted bacterial genomic DNA of the replicate cultivation bottles in Experiment 1 was equally mixed and subjected to 16S rRNA gene amplicon sequencing [21,22]. For genomic DNAs of biological samples in Experiment 2, individual sample units (i.e., animals) were retained, with at least *n* = 3 samples per treatment. The primer set of the first PCR was 515F (5′-GTGCCAGCMGCCGCGGTAA-3′) and 806R (5′-GGACTACHVHHHTWTCTAAT-3′), and the 5′-end was connected to the universal tag sequence for the following PCR. The T100 thermal cycler (Bio-Rad Inc.) and Ex Taq (Takara Bio Inc.) were used to generate the amplicons. The thermal cycler conditions used for amplification were as follows: initial denaturation at 95 °C for 10 s, and 25 cycles of 95 °C for 10 s, 57 °C for 30 s, and 72 °C for 30 s for the first PCR to generate the amplicons with the universal tag for the second PCR primer sets, and 10 cycles of 95 °C for 10 s, 57 °C for 30 s, and 72 °C for 30 s were used for the second PCR to obtain barcoded amplicons suitable for an Illumina MiSeq platform (Illumina, San Diego, CA, USA), according to manufacturer’s instructions using the 2  ×  250 bp paired-ends protocol. The second amplicons were subjected to paired-end sequencing. Microbial community analysis was conducted based on amplicon sequence variants (ASVs), and clean tags were denoised using the DADA2 plug-in in QIIME2 software (2026.04 Release) [22]. After filtering, quality control, and chimera removal, the average number of reads was calculated. The rarefaction line in all samples extended to the right end of the axis and flattened. The sequencing data were deposited in the DNA Data Bank of Japan Sequence Read Archive under BioProject accession number SSUB046790.

Fermentation parameter percentage change and genus-level log2 fold-change heatmap were generated with Jung et al. [25]. The formula was as follows:

$$\mathrm{Percentage}\;\mathrm{change}\left(\%\right)=\frac{{\mathrm{Mean}}_{\mathrm{treatment}}-{\mathrm{Mean}}_{\mathrm{control}}}{{\mathrm{Mean}}_{\mathrm{control}}}\times 100$$

$$\log_{2}FC=\log_{2}\left(\frac{\mathrm{Mean}\;{\mathrm{abundance}}_{\mathrm{treatment}}}{\mathrm{Mean}\;{\mathrm{abundance}}_{\mathrm{control}}}\right)$$

### 2.4. Statistical Analysis

#### 2.4.1. Experiment 1

The in vitro fermentation parameters and microbial community relative abundance data were analyzed using a linear mixed model, with treatment as a fixed effect and donor goat as a random effect. The statistical model was:

$$Y_{ijk}=\mu +T_{i}+D_{j}+\varepsilon_{ijk}$$

where *Y_ijk_* is the observed value of the dependent variable, μ is the overall mean, *T_i_* is the fixed effect of the treatment, *D_j_* is the random effect of the donor goat, and ε*_ijk_* is the residual error associated with the kth bottle observation within the donor goat and treatment.

Relative abundance and diversity analyses were conducted after total sum scaling (TSS) normalization. Alpha diversity indices (including Ace, Chao 1, Shannon, and Simpson) were used to analyze species diversity within samples. Principal coordinate analysis (PCoA) based on the Bray–Curtis distance was used to evaluate the significance of community structure differences. LEfSe analysis (LDA > 2, *p* < 0.05) was performed to identify discriminatory bacterial taxa at the family and genus levels, the Kruskal–Wallis test was utilized to identify species showing significant abundance differences among groups, and the Wilcoxon rank-sum test was used to compare differences between groups. PICRUSt2 was used to predict potential microbial functional profiles based on the KEGG database.

#### 2.4.2. Experiment 2

For the 3 × 3 Latin square feeding trial, ex vivo rumen fermentation parameters, fecal parameters, growth-related results, and microbial community relative abundance data were analyzed using a linear mixed model with treatment and period as fixed effects and animal as a random effect. The statistical model was:

$$Y_{ijk}=\mu +T_{i}+P_{j}+A_{k}+\varepsilon_{ijk}$$

where *Y_ijk_* is the observed value of the dependent variable, μ is the overall mean, *T_i_* is the fixed effect of treatment, *P_j_* is the fixed effect of period, *A_k_* is the random effect of animal, and *ε_ijk_* is the residual error. When a significant treatment effect was detected, pairwise comparisons among treatment means were performed based on estimated marginal means using the least-significant-difference (LSD) test. The results are presented as estimated marginal means with their standard errors. Statistical significance was declared at *p* < 0.05, and 0.05 ≤ *p* < 0.10 was considered a tendency. All statistical analyses were performed using IBM SPSS Statistics version 27.0 (IBM Corp., Armonk, NY, USA).

The alpha diversity, beta diversity, and PICRUSt2 analysis were performed in the same manner as those for Experiment 1. Mantel tests and co-occurrence network analyses were performed in R to explore associations between microbial community structures, fermentation-related variables, and potential microbial interaction patterns. The Mantel tests were based on the Bray–Curtis distances of ASV-level communities and Euclidean distances of fermentation parameters, using Spearman correlations with 999 permutations. Genus-level co-occurrence networks were constructed using Spearman correlations, retaining significant edges with |r| ≥ 0.85 and *p* ≤ 0.05, and network topology was calculated using the igraph package (version 2.2.2) and visualized in Gephi (version 0.11.2). Modules were detected using the modularity function in Gephi. For taxa without clear genus-level annotation, non-informative terms, such as “unclassified” and “Incertae Sedis”, were removed from node labels, and the nearest available higher-level taxonomic annotation was retained for visualization.

## 3. Results

### 3.1. Experiment 1 (In Vitro Experiment)

#### 3.1.1. Effects of ST and PE on In Vitro Fermentation Parameters

The addition of ST at 10^4^ and 10^6^ significantly increased the total gas production (Table 1) and methane proportion. The addition of ST at 10^8^ significantly increased the abundance of archaea. The isolate significantly increased the total VFA concentration and PA%, and decreased AA% and the A/P ratio compared with the control. ST significantly increased ammonia nitrogen at a supplementation concentration of 10^8^.

PE supplementation significantly increased the total gas production at all incubation levels (Table 2). PE supplementation at 10^4^ resulted in a lower methane proportion than that observed at PE supplementation of 10^6^ and 10^8^, whereas all supplementation levels significantly increased methane production. Compared with the control, supplementation with isolate B at 10^4^ and 10^6^ significantly decreased the amount of bacteria, whereas all supplementation levels decreased the amount of archaea. All PE inclusion levels showed significant increases in PA% and decreased the A/P ratio compared with the control.

#### 3.1.2. Effect of ST and PE on the In Vitro Fermentation Microbial Community

There were no significant differences between the alpha diversity (Supplementary Tables S1 and S2) and beta diversity based on the Bray–Curtis distance among treatment groups (Supplementary Figure S1).

As the amount of ST added increased, the relative abundance of *Xylanibacter* decreased and became significantly lower than that of the control. The relative abundance of Oscillospiraceae_UCG-002 decreased with increasing addition of ST. The relative abundances of *Fibrobacter* and Clostridia vadinBB60 group_Incertae Sedis family_Incertae Sedis increased with increasing inclusion of ST, which were significantly higher than those of the control (Table 3). The relative abundance of Prevotellaceae significantly decreased with ST supplementation, whereas those of Fibrobacteraceae, Clostridia vadinBB60 group_Incertae Sedis, and Acholeplasmataceae significantly increased with ST supplementation (Table 3). At the family level, Bacteroidales F082 and Spirochaetaceae were significantly affected by PE. At the genus level, the relative abundance of Bacteroidales_F082_Incertae Sedis decreased with supplementation of PE at a concentration of 10^6^ and was significantly lower than that of the control. The relative abundance of Prevotellaceae UCG-003 was significantly higher than that of the control at a PE concentration of 10^6^ (Table 4).

### 3.2. Experiment 2 (Feeding Experiment)

#### 3.2.1. Effect of Supplementary Isolates on Animal Performance, Rumen and In Vitro Fermentation Parameters

Supplementation with rumen-derived isolates did not significantly affect rumen fermentation parameters (Table 5) and animal performance parameters (Supplementary Table S3). After 24 h of incubation of isolates, PA% was significantly increased, whereas BA% was significantly decreased after the incubation of isolates (Table 6). The addition of isolates significantly decreased the amounts of archaea and ammonia nitrogen, and the lowest amounts of archaea and ammonia nitrogen were observed with PE addition. Regarding fecal parameters, the addition of isolates significantly increased the total VFA concentration and abundance of archaea (Table 7).

#### 3.2.2. Effect of Supplementary Isolates on Microbial Community

The amplicon sequencing of 16S rRNA genes revealed a total of 11,467,327 non-chimeric sequences. No significant differences in alpha (Chao1, Shannon, Ace, and Simpson, Supplementary Tables S4–S6) and beta diversity indices were observed using principal coordinate analysis (PCoA) based on Bray–Curtis distance (Supplementary Figure S1). Isolate supplementation did not significantly affect the relative abundance of most microbial taxa in the original rumen fluid, ex vivo rumen fermentation, or fecal samples (Table 8, Table 9 and Table 10). In the original rumen fluid, Bacteroidota, Bacillota, and Verrucomicrobiota were the dominant phyla. The only change was that ST supplementation significantly decreased the relative abundance of Prevotellaceae UCG-003 compared with the control.

The microbial composition in the in vitro rumen fermentation samples showed patterns generally similar to those observed in the original rumen fluid (Table 9). Compared with the control, the isolates tended to exhibit higher relative abundances of *Anaeroplasma* and Pseudomonadota. The taxonomic composition of the fecal samples tended to be different from that of the rumen-associated samples (Table 10), which was also shown by PCoA (Supplementary Figure S1). In fecal microbial communities, Bacillota dominated the bacterial community, followed by Bacteroidota. Compared with the control, isolates tended to exhibit lower relative abundances of *Alistipes* and higher relative abundances of *Butyrivibrio*.

#### 3.2.3. Effect of Supplementary Isolates on Directional Changes Relative to the Control

**Figure 1:**
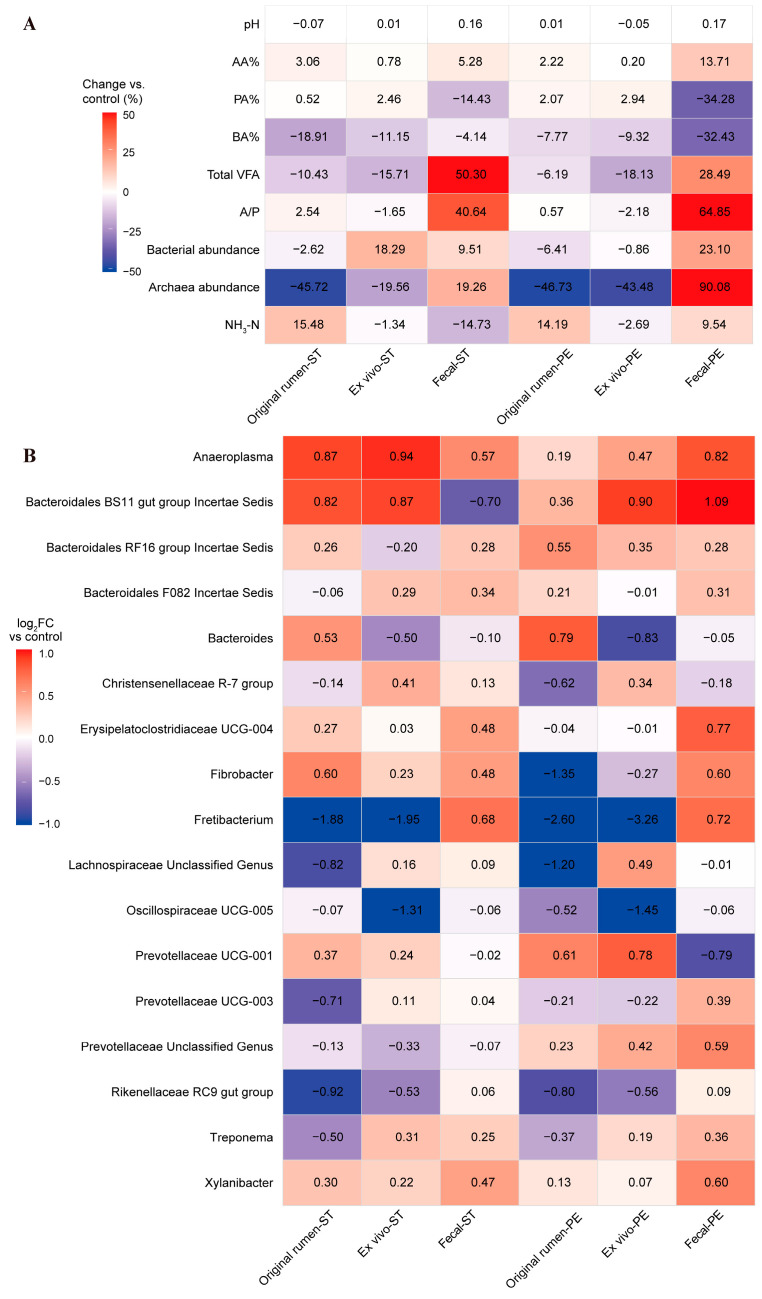
(**A**) Percentage changes in fermentation-related variables relative to the control; (**B**) Log2 fold changes in selected bacterial genera relative to the control.

A heatmap was used to summarize the directional changes in isolates relative to the control across the three systems (Figure 1). Overall, the response patterns in fecal samples differed from those in the rumen-associated systems. The original rumen fluid and ex vivo rumen fermentation results showed similar numerical patterns, with lower archaeal abundance and total VFA concentration in the isolates compared with the control. In contrast, these variables showed higher values in fecal samples after supplementation with isolates. The isolates exhibited higher relative abundances of *Anaeroplasma* and *Xylanibacter*, and lower relative abundances of Oscillospiraceae UCG-005 across all systems compared with the control. In the rumen-associated systems, the isolate groups exhibited lower relative abundances of Fretibacterium and Rikenellaceae RC9 gut group, whereas their relative abundances were higher in fecal samples. In contrast, the abundance of Prevotellaceae UCG-001 was higher in the rumen-associated samples but lower in fecal samples after isolate supplementation. Compared with the control, ST increased the relative abundance of *Fibrobacter* across all systems and decreased the relative abundance of Prevotellaceae_unclassified_Genus. However, PE showed an opposite pattern for these taxa: Prevotellaceae_unclassified_genus was increased, whereas *Fibrobacter* was decreased compared with that in the control in the rumen-associated systems, but higher in fecal samples.

#### 3.2.4. Effect of Supplementary Isolates on Mantel Test and Co-Occurrence Network Analysis

Mantel tests were performed to evaluate the associations between bacterial community composition and fermentation parameters or selected genera in different isolate groups. Bacterial community variation was not significantly associated with bacterial or archaeal abundance in any treatment group. No significant associations were also observed between bacterial community variation and selected genera, including Prevotellaceae UCG-003, *Fibrobacter*, *Fretibacterium*, *Anaeroplasma*, and Prevotellaceae UCG-001. Compared with the control group, the isolate groups exhibited significant associations between bacterial community variation and *Treponema*. In the ST group, bacterial community variation was significantly associated with Rikenellaceae RC9 gut group, suggesting that this taxon may be related to cross-system microbial community variations. In the PE group, bacterial community variations were significantly associated with the total VFA concentration, suggesting that total VFAs may be associated with microbial community structure under PE supplementation.

**Figure 2:**
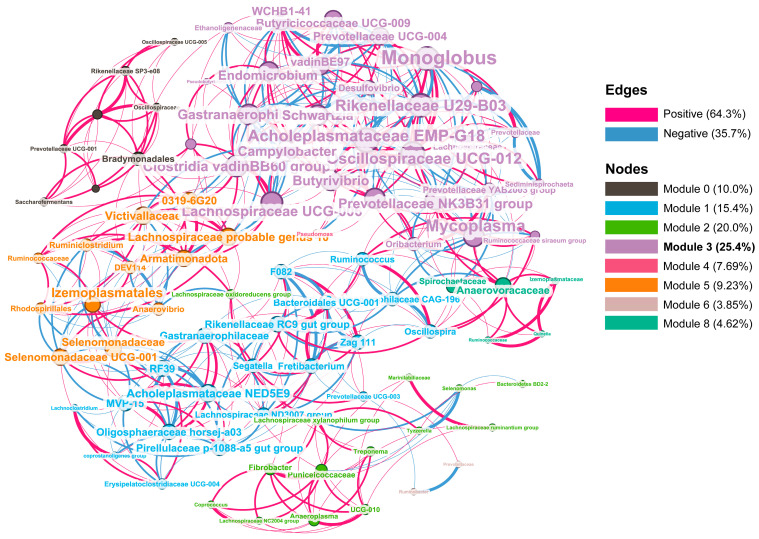
Genus-level microbial co-occurrence networks in control. A connection represents a significant Spearman correlation between bacterial genera with an absolute correlation coefficient ≥ 0.85 and *p*-value ≤ 0.05. Red and blue edges represent positive and negative correlations, respectively, and edge thickness is proportional to the absolute correlation coefficient. Nodes represent bacterial genera, node size is proportional to node degree, and node color corresponds to the modularity class calculated in Gephi. Nodes were filtered based on degree; nodes with degree values below 5 were not displayed, and only modules present in the displayed network are shown in the legend.

**Figure 3:**
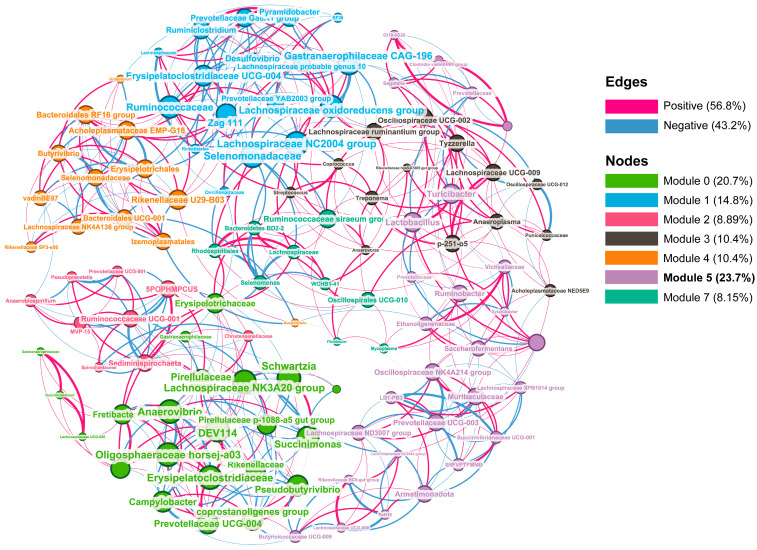
Genus-level microbial co-occurrence networks in PE. A connection represents a significant Spearman correlation between bacterial genera with an absolute correlation coefficient ≥ 0.85 and *p*-value ≤ 0.05. Red and blue edges represent positive and negative correlations, respectively, and edge thickness is proportional to the absolute correlation coefficient. Nodes represent bacterial genera, node size is proportional to node degree, and node color corresponds to the modularity class calculated in Gephi. Nodes were filtered based on degree; nodes with degree values below 5 were not displayed, and only modules present in the displayed network are shown in the legend.

**Figure 4:**
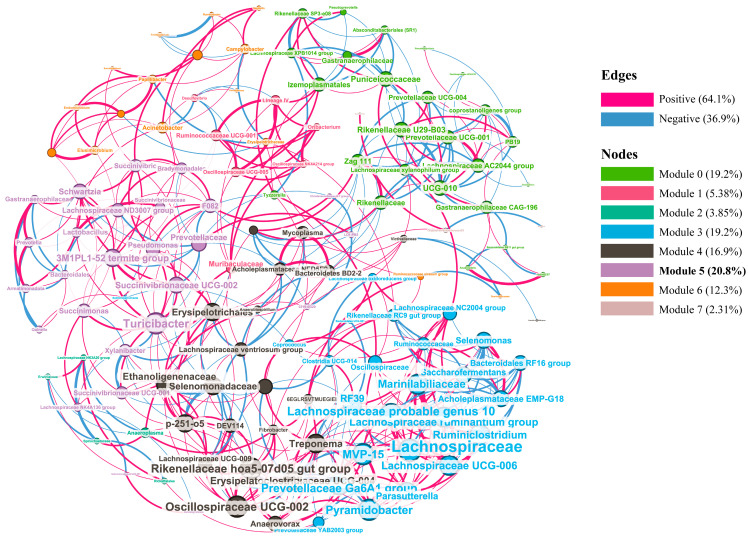
Genus-level microbial co-occurrence networks in ST. A connection represents a significant Spearman correlation between bacterial genera with an absolute correlation coefficient ≥ 0.85 and *p*-value ≤ 0.05. Red and blue edges represent positive and negative correlations, respectively, and edge thickness is proportional to the absolute correlation coefficient. Nodes represent bacterial genera, node size is proportional to node degree, and node color corresponds to the modularity class calculated in Gephi. Nodes were filtered based on degree; nodes with degree values below 5 were not displayed, and only modules present in the displayed network are shown in the legend.

Co-occurrence network analysis was performed to further evaluate bacterial association patterns in rumen-related systems (Table 11, Figure 2, Figure 3 and Figure 4). The PE group had a higher modularity value (0.689) than the other two. The composition of the largest module in the co-occurrence network differed among groups. In the control group, the dominant module contained 33 nodes, accounting for 25.4% of all nodes. In the ST group, the dominant module contained 27 nodes, accounting for 20.8% of all nodes. In the PE group, the dominant module contained 32 nodes, accounting for 23.7% of all nodes. Compared with the Control network, the ST network showed fewer edges, a lower average degree, and lower graph density, whereas the PE network showed a higher proportion of negative associations and a higher modularity index.

## 4. Discussion

### 4.1. Effects of Isolates on Preliminary In Vitro Rumen Fermentation and Microbial Community

In vitro incubation using rumen fluid from different individual goats demonstrated that both isolates significantly affected several rumen fermentation parameters, and their effects varied between different supplementation doses. After 24 h of incubation, both isolates significantly increased PA% and decreased A/P and were preferable in a more energetically favorable fermentation pathway associated with improved energy efficiency in ruminants. Previous studies have suggested that LAB introduced into the rumen may promote the growth of lactate-utilizing bacteria, thereby converting lactate into propionate [26]. This may partly explain the increased PA% observed after ST supplementation. In particular, *Selenomonas* can utilize soluble carbohydrates and lactate [27], and its relative abundance was significantly increased in the ST group in this study. The results observed in this study may improve fermentation efficiency. Supplementation with ST significantly increased the proportion of methane at all doses, and the total VFA concentration also increased significantly in a dose-dependent manner. This suggests that ST may have enhanced the overall rumen fermentation activity and promoted the formation of fermentation end products. The effects of ST were more pronounced at the high dose (10^8^ CFU/mL), under which archaeal abundance was significantly increased and total gas production was decreased. ST may have affected the microbial community mainly through substrate competition and the competitive exclusion of microorganisms with similar substrate preferences. This may explain why ST, especially at the high dose, decreased the relative abundances of *Prevotellaceae* and *Xylanibacter*, which are capable of utilizing various proteins and polysaccharides [28]. In contrast, ST increased the relative abundance of *Succinivibrio dextrinosolvens*, which can utilize intermediate substrates such as maltose [29]. The main substrates utilized by *Fibrobacteraceae* are cellulose and hemicellulose [30,31], which do not overlap with the substrate preference of *Streptococcus*. ST may have reduced the activity or relative abundance of indigenous bacteria through competitive interactions [27], and favored fiber-degrading activity. Significant increases in the relative abundances of Fibrobacteraceae, *Fibrobacter*, and *Fibrobacter succinogenes* were observed after ST supplementation, whereas the fermentation product excluded molecular hydrogen. Generally, cellulose fermentation and hemicellulose digestion can generate hydrogen, carbon dioxide, and soluble sugars [28], which are involved in archaeal methanogenesis in the rumen. This may further explain the increased archaeal abundance and methane percentage observed at the higher ST dosages, by stimulating other types of fibroclastic microorganisms such as protozoa.

The potential effects of *Pediococcus* sp. on ruminal fermentation characteristics were explored in previous studies that tested *Pediococcus* [32,33]. PE supplementation significantly increased methane production. PE, a type of LAB, ferments glucose mainly into lactic acid without producing hydrogen, which may decrease the availability of hydrogen for methanogenesis [34]. Several studies have also reported that LAB may suppress methane production [10,35,36]. The genome of *Pediococcus acidilactici* NRCC1 has been reported to encode multiple enzymes involved in the utilization of different carbohydrates [37], suggesting broad sugar-utilization capacity in *Pediococcus*. Rabee et al. [28] also reported that non-cellulolytic bacteria are important for utilizing soluble sugars released by cellulose hydrolysis, thereby preventing feedback inhibition of cellulolytic bacteria. Low-dose supplementation may have enhanced the utilization of soluble carbohydrates and intermediate products of carbohydrate degradation, producing a synergistic effect with fiber-degrading bacteria. However, at higher doses, PE may have had a slightly greater effect; overall, the fermentation characteristics showed a similar tendency to mid (10^6^)-level inoculation. Rather, high-dose PE supplementation significantly decreased the relative abundance of Spirochaetaceae, indicating that excessive supplementation may have suppressed some fiber-degrading bacteria. Considering competition for energy sources, PE may exclusively target other rumen bacteria. The antimicrobial metabolites produced by LAB can exert broad-spectrum inhibitory effects [38], and methanogenic archaea are sensitive to changes in pH. However, significant decreases in bacterial and archaeal abundances were observed in this study. Low-dose PE supplementation may represent a more balanced strategy for modulating rumen fermentation; therefore, this interpretation was valuable for the subsequent validation using animals.

### 4.2. Effects of Continuous Supplementation Isolates on In Vivo Rumen Fermentation (Original Rumen and Ex Vivo Fermentation)

Continuous supplementation with the isolates did not alter the basal fermentation status of rumen fluid, and the two isolates showed generally similar trends after continuous feeding. Previous studies have demonstrated that DFM supplementation can affect rumen fermentation and microbial communities, but long-term feeding may produce different responses than short-term in vitro incubation [18,39,40]. The aspects examined via in vivo rumen communities and in vitro fermentation fundamentally differed. Therefore, the fermentation responses observed in the adapted rumen fluid may have been jointly affected by the basal status of the rumen fluid and the remaining effects of isolate supplementation on the in vitro fermentation potential. In this regard, specific in vitro fermentation using rumen fluid collected from goats after isolate supplementation, namely ex vivo fermentation, might provide a more comprehensive overview of microbial and fermentation changes [39]. The effects of isolates on rumen fermentation under in vivo feeding conditions should be further investigated. In the ex vivo fermentation using rumen fluid collected after continuous supplementation, isolate supplementation significantly increased PA%, decreased BA%, and decreased the abundance of archaea. These responses were partially consistent with the results obtained using non-adapted rumen fluid in the preliminary in vitro incubation. The rumen fluid source can affect the response of in vitro rumen fermentation to probiotic supplementation, and rumen fluid from adapted and non-adapted animals may respond differently [40]. The effects of isolate supplementation were more pronounced in non-adapted rumen fluid than in rumen fluid collected after continuous supplementation. Oyebade et al. [39] observed that DFM supplementation improved fiber digestibility when non-adapted rumen fluid was used; however, this effect was not observed when rumen fluid collected from DFM-consuming cows was used as the inoculum for the ex vivo batch culture. Rossi, et al. [41] also reported that additives showing short-term activity in vitro did not significantly alter fermentation parameters of rumen fluid after long-term supplementation for two weeks. Kumar et al. [18] studied long-term supplementation with *Streptococcus gallolyticus* strain TDGB 406 and found that DFM may modify specific functional groups without markedly changing the overall rumen environment. The supplementation with isolates may not only exert immediate effects during short-term in vitro incubation but also alter the basal status and subsequent fermentation potential of rumen fluid after continuous feeding, presumably due to modification of the rumen microbial environment by the continuous supplementation. The results of this study suggested that the adapted rumen fluid did not influence any of the fermentation parameters compared with non-adapted rumen fluid, and that prior exposure may alter the response potential of rumen fluid modification, rather than directly change its basal fermentation profile, in accordance with previous findings [40].

The absence of significant differences in alpha and beta diversity did not necessarily indicate that microbial interactions remained unchanged—the microbial communities may have still differed in their co-occurrence network structures [42]. As the original rumen fluid and subsequent in vitro rumen fermentation samples largely overlapped in the PCoA analysis, these two rumen-associated systems were combined to further evaluate isolate-associated changes in microbial co-occurrence networks. Based on correlation analysis, the associations shown in the networks should be interpreted as potential co-variation patterns rather than direct microbial interactions [43], Results could be regarded as exploratory evidence of microbial ecological restructuring. Compared with the control group, isolate supplementation altered the association patterns between microbial taxa and fermentation parameters, suggesting that the isolates may have reshaped the fermentation–microbiota interaction network, rather than simply increasing or decreasing individual microbial taxa. In particular, it was speculated that ST resulted in fewer edges, a lower average degree, and lower graph density, and PE resulted in the highest modularity value (0.689) compared with the other two groups. This suggests that microbial taxa in the PE group may have formed more clearly separated modules [44], which may be associated with increased network stability in previous ecological studies [45]; however, network stability was not directly evaluated in the present study. The PE network showed a higher proportion of negative associations, suggesting potential competitiveness with some inhabiting bacteria [45]. This implication is in accordance with the preceding description that microbial taxa occupying similar ecological niches may exhibit mutual exclusion because they compete for limited nutrients and ecological space [46].

Across the two experiments and different systems, ST showed a relatively consistent tendency to increase the relative abundance of *Fibrobacter*. In the ST group, the *Fibrobacter*-containing module, Module 4, exhibited an altered composition and direction of co-variation compared with the control network. Setting aside *Fibrobacter*, Rikenellaceae RC9 gut group, Rikenellaceae hoa5-07d05 gut group, Lachnospiraceae unclassified genus, *Anaeroplasma*, Oscillospiraceae UCG-002, *Anaerovorax*, and *Anaerobiospirillum*, all of which play roles in degrading plant-derived polysaccharides [47,48,49,50]. This positive correlation may indicate a change in the co-variation network to a more connected fiber-digestion-associated bacterial community.

On the other hand, in the PE group, the largest module, Module 5, contained 32 genera and included *Lactobacillus,* a major lactate-producing genus [51], multiple carbohydrate- and polysaccharide-utilizing taxa, Prevotellaceae UCG-003, *Prevotella*, Prevotellaceae Unclassified_Genus, *Segatella*, and *Xylanibacter*, which have been associated with the utilization of plant cell walls, as well as plant energy-storage polysaccharides [52,53]. Other bacteria representative in the rumen, such as Ruminococcaceae, Lachnospiraceae, and Rikenellaceae, which are involved in the degradation of structural carbohydrates and starch in the rumen of cows [54,55]. Acid-producing bacteria, including Succinivibrionaceae spp., which contribute to the production of succinate [56], were also clustered under PE addition. PE supplementation may have promoted the co-variation in lactate-producing bacteria with carbohydrate-utilizing taxa; however, as PE addition may have affected these modules in a competitive manner, it may be practically important to reduce the supplementation amount compared with ST to minimize its impact.

### 4.3. Limitations and Future Perspectives

The combined in vitro and in vivo experiments showed that the isolates affected in vitro rumen fermentation and microbial communities, and changed the microbial community network. The overall direction of these effects was partly consistent between in vitro incubation and the feeding trial. Nevertheless, several limitations should be acknowledged. The preliminary in vitro experiment evaluated the effects of the isolates only at the 24 h endpoint and did not assess temporal fermentation dynamics. In addition, the small number of experimental goats limited the statistical power of the feeding trial. Future studies should include more comprehensive experimental designs, such as continuous gas production monitoring or additional incubation time points, as well as feeding trials with a larger number of animals. Furthermore, even under identical feeding conditions, different community structures among animals may restrict the generalizability of the findings.

## 5. Conclusions

Two LAB species (ST and PE) isolated from native Japanese goats exerted differential effects on in vitro rumen fermentation and further altered the in vivo rumen environment after long-term supplementation in goats. Long-term supplementation with both isolates changed the basal fermentation status of rumen fluid, and the two isolates exhibited generally similar trends after continuous feeding. Rumen-derived bacterial isolates reshaped microbial interaction networks and consistently altered fermentation potential, despite limited effects on conventional in vivo fermentation traits due to continuous supplementation, which redirects the fermentation pathway of substrate digestion in the rumen. These findings highlight the importance of using microbial additives in ruminants and are important for understanding isolate-associated modulation of gastrointestinal fermentation, particularly regarding the effects on young ruminants, which are warranted to accumulate performance results from long-term feeding experiments across growth stages.

## Figures and Tables

**Table 1:** Effect of different doses of *Streptococcus* sp. isolate (ST) on in vitro fermentation parameters.

	Control	ST 10^4^	ST 10^6^	ST 10^8^	SEM	*p*-Value
pH	6.382	6.517	6.502	6.430	0.056	0.074
Total gas production (mL/g substrate)	142.7 ^b^	153.5 ^a^	142.9 ^b^	138.0 ^b^	5.509	0.023
CH_4_ (%)	15.89 ^b^	16.08 ^b^	16.61 ^a^	16.66 ^a^	0.963	0.006
CH_4_ production (mL/g substrate)	22.56	24.58	23.73	22.86	0.805	0.100
Bacteria (×10^7^ copies/mL)	167.5	179.9	226.2	206.2	40.178	0.051
Archaea (×10^7^ copies/mL)	2.619 ^bc^	2.181 ^c^	3.524 ^ab^	4.007 ^a^	0.574	0.021
Total VFA (μmol/mL)	63.06 ^b^	67.42 ^a^	69.25 ^a^	69.40 ^a^	5.544	0.013
Acetate (mol%)	57.67 ^a^	56.37 ^b^	56.39 ^b^	56.24 ^b^	2.111	0.000
Propionate (mol%)	19.93 ^b^	20.66 ^a^	20.83 ^a^	20.98 ^a^	0.939	0.000
Butyrate (mol%)	22.40	22.97	22.78	22.78	2.082	0.177
A/P	2.902 ^a^	2.746 ^b^	2.727 ^b^	2.691 ^b^	0.181	0.000
NH_3_-N (mg/100 mL)	13.63 ^b^	13.58 ^b^	13.30 ^b^	15.06 ^a^	0.406	0.025

ST, *Streptococcus* sp. isolate; ST 10^4^, ST 10^6^, and ST 10^8^ indicate supplementation levels of 10^4^, 10^6^, and 10^8^ CFU/mL, respectively; CH_4_, methane; VFA, volatile fatty acids; A/P, acetate-to-propionate ratio; NH_3_-N, ammonia nitrogen; SEM, standard error of the mean. Within a row, values with different superscript letters differ significantly (*p* < 0.05).

**Table 2:** Effect of different doses of *Pediococcus* sp. isolate (PE) on in vitro fermentation parameters.

	Control	PE 10^4^	PE 10^6^	PE 10^8^	SEM	*p*-Value
pH	6.517	6.478	6.470	6.457	0.051	0.250
Total gas production (mL/g substrate)	142.5 ^b^	162.3 ^a^	156.7 ^a^	160.1 ^a^	27.78	<0.001
CH_4_ (%)	17.29 ^ab^	16.82 ^b^	17.98 ^a^	18.05 ^a^	1.105	0.033
CH_4_ production (mL/g substrate)	24.64 ^b^	27.35 ^a^	28.23 ^a^	28.89 ^a^	5.193	0.003
Bacteria (×10^7^ copies/mL)	256.5 ^a^	215.4 ^b^	160.8 ^c^	227.3 ^ab^	47.40	<0.001
Archaea (×10^7^ copies/mL)	3.072 ^a^	2.284 ^b^	0.948 ^c^	2.056 ^b^	0.560	<0.001
Total VFA (μmol/mL)	59.68	60.87	62.45	60.70	8.315	0.366
Acetate (mol%)	56.74	55.77	56.06	54.57	3.003	0.061
Propionate (mol%)	22.50 ^b^	23.50 ^a^	23.46 ^a^	23.13 ^a^	1.570	0.010
Butyrate (mol%)	20.76	20.73	20.48	22.30	1.985	0.259
A/P	2.554 ^a^	2.406 ^b^	2.425 ^b^	2.400 ^b^	0.273	<0.001
NH_3_-N (mg/100 mL)	13.87	13.90	14.08	14.04	0.117	0.462

PE, *Pediococcus* sp. isolate; PE 10^4^, PE 10^6^, and PE 10^8^ indicate supplementation levels of 10^4^, 10^6^, and 10^8^ CFU/mL, respectively; CH_4_, methane; VFA, volatile fatty acids; A/P, acetate-to-propionate ratio; NH_3_-N, ammonia nitrogen; SEM, standard error of the mean. Within a row, values with different superscript letters differ significantly (*p* < 0.05).

**Table 3:** Effect of different doses of *Streptococcus* sp. isolate (ST) on bacterial relative abundance.

Family %	Control	ST 10^4^	ST 10^6^	ST 10^8^	SEM	*p*-Value
Prevotellaceae	27.57 ^a^	28.43 ^a^	27.52 ^a^	24.21 ^b^	5.677	0.015
Rikenellaceae	14.85	16.7	14.38	13.81	2.853	0.400
Bacteroidales F082	12.33	12.83	14.02	14.86	3.478	0.741
Bacteroidales BS11 gut group	7.696	6.843	6.517	7.522	2.085	0.270
Kiritimatiellia_WCHB1-41_Incertae Sedis	4.973	5.155	5.303	5.225	0.905	0.987
Oscillospiraceae	5.283	4.372	4.267	4.103	0.357	0.091
Fibrobacteraceae	1.335 ^b^	1.272 ^b^	1.402 ^b^	2.329 ^a^	0.463	0.010
Selenomonadaceae	0.908	0.977	1.296	1.124	0.415	0.090
Clostridia vadinBB60 group Incertae Sedis	0.752 ^b^	0.625 ^b^	0.831 ^b^	1.148 ^a^	0.225	0.005
Acholeplasmataceae	0.396 ^b^	0.433 ^b^	0.492 ^b^	0.701 ^a^	0.196	0.005
Genus %						
*Xylanibacter*	20.61 ^a^	20.71 ^a^	19.93 ^a^	17.72 ^b^	5.637	0.020
Bacteroidales_F082_Incertae Sedis	12.33	12.83	14.01	14.86	3.478	0.741
Rikenellaceae RC9 gut group	11.86	12.82	11.17	10.62	2.705	0.436
Oscillospiraceae_UCG-002	3.734 ^a^	3.150 ^b^	3.060 ^b^	2.635 ^b^	0.675	0.016
Prevotellaceae UCG-001	1.767	1.961	1.935	1.564	1.165	0.260
*Fibrobacter*	1.334 ^b^	1.271 ^b^	1.401 ^b^	2.328 ^a^	0.462	0.010
Prevotellaceae UCG-003	1.276	1.578	1.602	1.416	0.199	0.081
Clostridia vadinBB60 group_Incertae Sedis family_Incertae Sedis	0.752	0.625	0.831	1.148	0.225	0.005
*Segatella*	0.888	0.864	0.861	0.741	0.248	0.118
*Selenomonas*	0.249 ^b^	0.247 ^b^	0.408 ^a^	0.338 ^ab^	0.084	0.048
Species %						
*Fibrobacter succinogenes*	1.221 ^b^	1.123 ^b^	1.291 ^b^	2.169 ^a^	0.403	0.009
*Prevotella ruminicola*	0.505	0.579	0.506	0.484	0.235	0.543
*Anaeroplasma abactoclasticum*	0.234	0.296	0.315	0.365	0.180	0.136
*Treponema ruminis*	0.110	0.105	0.146	0.141	0.066	0.205
*Succinivibrio dextrinosolvens*	0.029 ^b^	0.044 ^ab^	0.035 ^b^	0.059 ^a^	0.021	0.031

ST, *Streptococcus* sp. isolate; ST 10^4^, ST 10^6^, and ST 10^8^ indicate supplementation levels of 10^4^, 10^6^, and 10^8^ CFU/mL, respectively; SEM, standard error of the mean. Within a row, values with different superscript letters differ significantly (*p* < 0.05).

**Table 4:** Effect of different doses of *Pediococcus* sp. isolate (PE) on bacterial relative abundance.

Family %	Control	PE 10^4^	PE 10^6^	PE 10^8^	SEM	*p*-Value
Prevotellaceae	30.75	31.04	37.47	32.23	5.082	0.166
Rikenellaceae	12.07	10.06	12.69	11.40	2.309	0.207
Bacteroidales_F082	9.714 ^a^	9.403 ^a^	8.678 ^b^	9.182 ^ab^	3.575	0.026
Bacteroidales BS11 gut group	6.895	7.563	5.287	7.573	2.976	0.349
Kiritimatiellia_WCHB1-41_Incertae Sedis	6.671	6.900	6.616	6.647	0.805	0.965
Bacteroidales RF16 group	4.195	4.240	4.944	4.372	1.114	0.156
Synergistaceae	3.647	3.832	0.769	3.252	1.535	0.110
Selenomonadaceae	1.831	2.468	2.022	2.027	1.437	0.460
Fibrobacteraceae	1.867	2.038	0.901	1.887	0.648	0.117
Spirochaetaceae	1.028 ^a^	0.684 ^b^	0.721 ^b^	0.679 ^b^	0.063	0.020
Genus %						
*Xylanibacter*	23.65	24.05	29.42	25.21	4.701	0.186
Rikenellaceae RC9 gut group	9.922	8.050	10.25	9.375	3.137	0.250
Bacteroidales_F082_Incertae Sedis	9.714 ^a^	9.403 ^a^	8.678 ^b^	9.182 ^ab^	3.575	0.026
Bacteroidales BS11 gut group_Incertae Sedis	6.895	7.563	5.287	7.573	2.976	0.349
*Fibrobacter*	1.863	2.037	0.901	1.887	0.648	0.118
Prevotellaceae UCG-003	1.098 ^b^	1.037 ^b^	1.281 ^a^	1.118 ^b^	0.167	0.030
*Ruminobacter*	1.057	0.919	1.305	0.993	0.691	0.147
*Segatella*	0.778	0.910	1.086	0.756	0.550	0.457
Species %						
*Fibrobacter succinogenes*	1.790	1.940	0.830	1.800	0.617	0.120
*Prevotella ruminicola*	0.477	0.411	0.608	0.455	0.223	0.487
*Treponema ruminis*	0.241 ^a^	0.169 ^b^	0.201 ^c^	0.170 ^bc^	0.065	0.004
*Anaeroplasma abactoclasticum*	0.096	0.110	0.123	0.131	0.069	0.511
*Succinivibrio dextrinosolvens*	0.088	0.102	0.129	0.114	0.040	0.170
*Ruminococcus albus*	0.040	0.052	0.034	0.032	0.016	0.622

PE, *Pediococcus* sp. isolate; PE 10^4^, PE 10^6^, and PE 10^8^ indicate supplementation levels of 10^4^, 10^6^, and 10^8^ CFU/mL, respectively; SEM, standard error of the mean. Within a row, values with different superscript letters differ significantly (*p* < 0.05).

**Table 5:** Rumen fermentation parameter of goats after long term supplementation with ST and PE.

Item	Control	ST	PE	SEM	*p*-Value
pH	6.665	6.600	6.672	0.230	0.971
Acetate (mol%)	60.84	62.70	62.19	0.459	0.186
Propionate (mol%)	28.55	28.70	29.14	0.666	0.824
Butyrate (mol%)	10.61	8.603	9.785	0.809	0.391
Total VFA (μmol/mL)	66.61	59.67	62.49	11.30	0.913
A/P	2.132	2.186	2.144	0.057	0.802
Bacteria (×10^7^ copies/mL)	702.5	684.0	657.4	107.8	0.958
Archaea (×10^7^ copies/mL)	2.719	1.476	1.448	0.476	0.301
NH_3_-N (mg/100 mL)	4.910	5.670	5.607	0.399	0.472

ST, *Streptococcus* sp. isolate; PE, *Pediococcus* sp. isolate; VFA, volatile fatty acids; A/P, acetate-to-propionate ratio; NH_3_-N, ammonia nitrogen; SEM, standard error of the mean.

**Table 6:** Ex vivo fermentation parameter of isolates on long term isolates supplementation adapted rumen fluid.

Item	Control	ST	PE	SEM	*p*-Value
pH	6.583	6.594	6.537	0.061	0.291
Digestibility (%)	54.73	62.31	53.98	3.335	0.159
CH_4_ (mL/g substrate)	19.34	18.51	19.34	0.492	0.314
CH_4_ (%)	12.91	12.69	12.82	0.251	0.691
Total gas production (mL/g substrate)	149.89	145.99	150.62	4.232	0.253
Acetate (mol%)	58.98	59.44	59.10	0.838	0.387
Propionate (mol%)	30.22 ^b^	30.96 ^a^	31.10 ^a^	0.606	0.008
Butyrate (mol%)	10.80 ^a^	9.596 ^b^	9.795 ^b^	0.486	0.007
Total VFA (μmol/mL)	84.84	71.51	69.46	5.527	0.126
A/P	1.954	1.921	1.911	0.060	0.136
Bacteria (×10^7^ copies/mL)	415.6	491.6	412.1	62.66	0.287
Archaea (×10^7^ copies/mL)	13.53 ^a^	10.88 ^b^	7.648 ^c^	2.526	<0.001
NH_3_-N (mg/100 mL)	5.647 ^a^	5.571 ^b^	5.495 ^c^	0.038	<0.001

ST, *Streptococcus* sp. isolate; PE, *Pediococcus* sp. isolate; CH_4_, methane; VFA, volatile fatty acids; A/P, acetate-to-propionate ratio; NH_3_-N, ammonia nitrogen; SEM, standard error of the mean. Within a row, values with different superscript letters differ significantly (*p* < 0.05).

**Table 7:** Effects of isolate supplementation on fecal parameters.

Item	Control	ST	PE	SEM	*p*-Value
pH	7.804	7.930	7.939	0.082	0.384
Moisture (%)	51.13	49.18	50.15	3.428	0.769
Acetate (mol%)	71.09	74.84	81.48	3.309	0.097
Propionate (mol%)	25.08	21.64	16.22	3.581	0.192
Butyrate (mol%)	3.749	3.439	2.460	1.615	0.471
Total VFA (μmol/g)	31.63 ^b^	47.21 ^a^	40.39 ^ab^	5.563	0.015
A/P	3.800	5.338	6.422	0.887	0.127
Bacteria (×10^7^ copies/g)	600.3	662.0	725.6	124.0	0.342
Archaea (×10^7^ copies/g)	0.350 ^b^	0.402 ^b^	0.660 ^a^	0.110	<0.001
NH_3_-N (mg/100 mL)	1.983	1.719	2.150	0.205	0.338

ST, *Streptococcus* sp. isolate; PE, *Pediococcus* sp. isolate; VFA, volatile fatty acids; A/P, acetate-to-propionate ratio; NH_3_-N, ammonia nitrogen; SEM, standard error of the mean. Within a row, values with different superscript letters differ significantly (*p* < 0.05).

**Table 8:** Effects of isolate supplementation on the relative abundance of original rumen microbial community.

	Control	ST	PE	SEM	*p*-Value
**Bacteroidota**	69.68	72.44	75.13	2.347	0.357
f__Prevotellaceae	38.15	42.34	41.58	5.106	0.833
g*__Xylanibacter*	26.73	32.81	29.33	3.036	0.442
s__*Prevotella ruminicola*	0.713	2.202	1.530	0.876	0.561
g__Prevotellaceae Unclassified_Genus	5.790	5.296	6.790	2.097	0.715
g__Prevotellaceae UCG-003	3.299 ^a^	2.011 ^b^	2.844 ^a^	0.571	0.019
g__Prevotellaceae UCG-001	0.765	0.989	1.168	0.381	0.770
g__*Segatella*	0.887	0.620	0.883	0.340	0.825
s__*Prevotella bryantii*	0.056	0.145	0.054	0.066	0.533
f__Bacteroidales RF16 group (g__Bacteroidales RF16 group Incertae Sedis)	8.775	10.498	12.832	2.387	0.467
f__Bacteroidales F082 (g__Bacteroidales F082 Incertae Sedis)	8.107	7.792	9.345	2.436	0.876
f__Rikenellaceae	10.175	5.788	6.272	2.335	0.456
g__Rikenellaceae RC9 gut group	9.402	4.962	5.392	2.264	0.399
**Bacillota**	16.898	13.696	13.262	2.247	0.477
f__Selenomonadaceae	3.349	1.396	2.254	0.847	0.404
g__*Selenomonas*	1.119	0.557	1.000	0.269	0.385
f__Lachnospiraceae	2.510	1.850	1.444	0.328	0.248
s__*Butyrivibrio fibrisolvens*	0.008	0.016	0.018	0.006	0.558
f__Clostridia vadinBB60 group (g__Clostridia vadinBB60 group Incertae Sedis)	1.268	1.127	1.040	0.138	0.551
f__Acholeplasmataceae	0.498	0.943	0.626	0.238	0.286
s__*Anaeroplasma abactoclasticum*	0.195	0.483	0.339	0.167	0.435
s__*Ruminococcus flavefaciens*	0.059	0.028	0.019	0.015	0.238
s__*Ruminococcus albus*	0.024	0.020	0.029	0.006	0.287
**Verrucomicrobiota**	4.491	4.745	4.331	0.806	0.661
f__Kiritimatiellia_WCHB1-41 Incertae Sedis (g__Kiritimatiellia_WCHB1-41 Incertae Sedis Incertae Sedis)	3.091	3.165	2.143	0.807	0.312
f__Victivallales vadinBE97 (g__Victivallales vadinBE97 Incertae Sedis)	1.108	1.164	1.699	0.171	0.126
**Cyanobacteriota**	3.452	3.838	3.521	0.533	0.830
f__Gastranaerophilaceae	3.448	3.832	3.514	0.534	0.832
g__Zag 111	0.985	1.180	1.114	0.220	0.824
g__CAG-196	0.918	1.401	0.951	0.269	0.465
**Pseudomonadota**	0.970	1.305	1.282	0.249	0.564
s__*Succinivibrio dextrinosolvens*	0.025	0.028	0.040	0.015	0.421
**Fibrobacterota**	1.047	1.587	0.410	0.298	0.115
f__Fibrobacteraceae	1.047	1.587	0.410	0.298	0.115
g_*_Fibrobacter*	1.047	1.587	0.410	0.298	0.114
s__*Fibrobacter succinogenes*	0.933	1.488	0.347	0.317	0.146
**Synergistota**	1.603	0.443	0.272	0.651	0.429
f__Synergistaceae	1.603	0.443	0.272	0.651	0.429
**Spirochaetota**	0.644	0.565	0.549	0.089	0.746
s__*Treponema ruminis*	0.054	0.020	0.017	0.025	0.572

ST, *Streptococcus* sp. isolate; PE, *Pediococcus* sp. isolate; SEM, standard error of the mean. Within a row, values with different superscript letters differ significantly (*p* < 0.05). Bold taxa indicate phylum-level classifications. f__, family; g__, genus; s__, species.

**Table 9:** Effects of isolate supplementation on the relative abundance of ex vivo microbial community.

	Control	ST	PE	SEM	*p*-Value
**Bacteroidota**	58.69	62.23	63.09	2.497	0.440
f__Prevotellaceae	31.78	34.83	34.96	4.395	0.828
g__*Xylanibacter*	21.90	25.60	22.94	2.539	0.437
s__*Prevotella ruminicola*	0.820	2.245	1.528	0.426	0.174
g__Prevotellaceae Unclassified_Genus	4.688	3.724	6.263	1.136	0.373
g__Prevotellaceae UCG-003	2.814	3.030	2.410	0.509	0.705
g__Prevotellaceae UCG-001	0.922	1.087	1.587	0.461	0.608
s__*Prevotella bryantii*	0.037	0.049	0.047	0.007	0.453
f__Rikenellaceae	9.683	7.588	7.124	1.063	0.301
g__Rikenellaceae RC9 gut group	8.623	5.991	5.834	1.305	0.337
f__Bacteroidales F082 (g__Bacteroidales F082 Incertae Sedis)	6.252	7.670	6.192	1.513	0.230
f__Bacteroidales RF16 group (g__Bacteroidales RF16 group Incertae Sedis)	5.196	4.521	6.616	1.178	0.484
**Bacillota**	16.14	15.80	17.08	0.317	0.163
f__Lachnospiraceae	3.274	3.137	3.630	0.540	0.810
g__Lachnospiraceae Unclassified_Genus	1.049	1.174	1.472	0.352	0.706
s__*Butyrivibrio fibrisolvens*	0.005	0.008	0.007	0.002	0.711
f__Acholeplasmataceae	1.829	3.425	2.683	0.435	0.101
g__*Anaeroplasma*	1.703	3.259	2.354	0.378	0.058
s__*Anaeroplasma abactoclasticum*	0.937	1.940	1.472	0.292	0.124
f__Oscillospiraceae	1.637	1.622	1.613	0.311	0.998
f__Clostridia vadinBB60 group (g__Clostridia vadinBB60 group Incertae Sedis)	1.458	0.846	1.040	0.274	0.365
s__*Ruminococcus albus*	0.036	0.038	0.065	0.024	0.553
s__*Ruminococcus flavefaciens*	0.034	0.025	0.016	0.011	0.519
**Verrucomicrobiota**	5.239	5.392	6.444	0.483	0.244
f__Kiritimatiellia_WCHB1-41 Incertae Sedis (g__Kiritimatiellia_WCHB1-41 Incertae Sedis Incertae Sedis)	3.721	3.718	4.442	0.360	0.323
f__Victivallales vadinBE97 (g__Victivallales vadinBE97 Incertae Sedis)	1.073	1.013	1.351	0.097	0.133
**Fibrobacterota**	5.607	6.562	4.641	0.591	0.186
f__Fibrobacteraceae	5.606	6.562	4.641	0.591	0.186
g__*Fibrobacter*	5.604	6.561	4.640	0.592	0.186
s__*Fibrobacter succinogenes*	5.278	6.335	4.503	0.625	0.230
**Cyanobacteriota**	3.329	3.257	3.000	0.402	0.838
f__Gastranaerophilaceae	3.323	3.250	2.995	0.403	0.839
**Synergistota**	6.448	1.732	0.757	3.232	0.494
f__Synergistaceae	6.448	1.732	0.757	3.232	0.494
g__*Fretibacterium*	6.404	1.661	0.668	3.251	0.494
**Pseudomonadota**	1.789	2.080	1.920	0.064	0.069
s__*Succinivibrio dextrinosolvens*	0.015	0.011	0.027	0.007	0.259
**Spirochaetota**	1.681	1.880	1.873	0.425	0.917
f__Spirochaetaceae	1.441	1.388	1.373	0.359	0.987
g__*Treponema*	1.018	1.264	1.160	0.307	0.847
s__*Treponema ruminis*	0.202	0.236	0.184	0.061	0.701
s__*Treponema porcinum*	0.031	0.048	0.044	0.009	0.478
s__*Treponema saccharophilum*	0.014	0.032	0.031	0.013	0.415

ST, *Streptococcus* sp. isolate; PE, *Pediococcus* sp. isolate; SEM, standard error of the mean. Bold taxa indicate phylum-level classifications. f__, family; g__, genus; s__, species.

**Table 10:** Effects of isolate supplementation on the relative abundance of fecal samples microbial community.

	Control	ST	PE	SEM	*p*-Value
**Bacillota**	51.02	51.00	48.69	2.731	0.486
f__Oscillospiraceae	13.21	12.70	12.40	0.856	0.572
g__Oscillospiraceae UCG-005	7.483	7.185	7.181	0.540	0.253
g__Oscillospiraceae UCG-002	1.620	1.532	1.486	0.088	0.595
f__Lachnospiraceae	9.460	10.01	9.359	0.852	0.078
g__Lachnospiraceae Unclassified_Genus	7.923	8.416	7.875	0.782	0.241
s__*Butyrivibrio fibrisolvens*	0.009 ^c^	0.015 ^a^	0.012 ^b^	0.006	0.007
f__Ruminococcaceae	4.157	4.178	3.842	0.507	0.733
s__*Ruminococcus flavefaciens*	0.077	0.077	0.053	0.029	0.796
f__Oscillospirales UCG-010 (g__Oscillospirales UCG-010 Incertae Sedis)	3.527	3.700	3.120	0.303	0.412
f__Christensenellaceae	2.736	2.998	2.463	0.776	0.219
g__Christensenellaceae R-7 group	2.636	2.891	2.324	0.740	0.201
f__Acidaminococcaceae	1.476	1.452	1.377	0.201	0.849
f__Monoglobaceae	1.358	1.314	1.466	0.110	0.659
g__*Monoglobus*	1.358	1.314	1.466	0.110	0.659
s__*Anaeroplasma abactoclasticum*	0.014	0.018	0.022	0.004	0.428
**Bacteroidota**	38.08	37.89	38.92	2.265	0.919
f__Rikenellaceae	11.29	10.73	10.93	1.392	0.937
g__Rikenellaceae RC9 gut group	7.416	7.734	7.883	1.372	0.945
g__*Alistipes*	2.676	1.878	2.166	0.175	0.054
f__Prevotellaceae	8.642	8.595	9.553	1.472	0.859
g__*Xylanibacter*	2.048	2.838	3.102	0.502	0.392
s__*Prevotella ruminicola*	0.048	0.065	0.078	0.014	0.421
g__Prevotellaceae UCG-003	1.799	1.844	2.358	0.491	0.556
g__Prevotellaceae UCG-004	2.278	1.510	1.654	0.358	0.367
s__*Prevotella bryantii*	0.046	0.064	0.072	0.013	0.431
f__Bacteroidaceae (g__Bacteroides)	7.742	7.233	7.456	0.482	0.769
Bacteroidales F082 (g__Bacteroidales F082 Incertae Sedis)	1.747	2.213	2.165	0.256	0.445
f__Bacteroidales Incertae Sedis	1.632	1.171	1.662	0.239	0.361
f__Bacteroidales RF16 group (g__Bacteroidales RF16 group Incertae Sedis)	1.301	1.578	1.582	0.297	0.700
**Verrucomicrobiota**	4.873	4.545	5.120	1.059	0.914
f__Akkermansiaceae	3.047	2.453	2.637	0.794	0.862
g__*Akkermansia*	3.047	2.453	2.637	0.794	0.862
f__Kiritimatiellia_WCHB1-41 Incertae Sedis	1.148	1.145	1.632	0.231	0.172
**Spirochaetota**	1.889	2.261	2.358	0.341	0.564
f__Spirochaetaceae	1.867	2.228	2.326	0.346	0.588
g__*Treponema*	1.698	2.017	2.176	0.328	0.562
s__*Treponema porcinum*	0.038	0.038	0.070	0.011	0.146
**Pseudomonadota**	1.256 ^c^	1.486 ^b^	2.130 ^a^	0.223	<0.001
**Cyanobacteriota**	0.906	0.942	0.972	0.144	0.948
**Thermodesulfobacteriota**	0.847	1.054	0.848	0.209	0.743
**Elusimicrobiota**	0.504	0.152	0.249	0.107	0.167
**Fibrobacterota**	0.127	0.178	0.193	0.024	0.248
s__*Fibrobacter succinogenes*	0.107	0.145	0.166	0.024	0.312

ST, *Streptococcus* sp. isolate; PE, *Pediococcus* sp. isolate; SEM, standard error of the mean. Within a row, values with different superscript letters differ significantly (*p* < 0.05). Bold taxa indicate phylum-level classifications. f__, family; g__, genus; s__, species.

**Table 11:** Topological properties of genus-level co-occurrence networks among treatment groups.

	Control	ST	PE
Modularity	0.632	0.633	0.689
Nodes	130	130	135
Edges	596	520	585
Positive edges (n)	383	328	332
Positive edges (%)	64.3	63.1	56.8
Negative edges (n)	213	192	253
Negative edges (%)	35.7	36.9	43.2
Modules (n)	9	8	9
Largest module	Module 3	Module 5	Module 5
Largest module nodes	33	27	32
Largest module (%)	25.4	20.8	23.7
Average degree	9.169	8.000	8.667
Graph density	0.071	0.062	0.065
Mean |r|	0.921	0.919	0.922

ST, *Streptococcus* sp. isolate; PE, *Pediococcus* sp. isolate. Genus-level co-occurrence networks were constructed based on Spearman correlation analysis. Edges represent significant correlations between bacterial taxa. Mean |r| indicates the average absolute Spearman correlation coefficient of the retained edges.

## Data Availability

He sequencing data were deposited in the DNA Data Bank of Japan Sequence Read Archive under BioProject accession number SSUB046790. All other experiment data will be open upon request.

## Supplementary Materials

The following supporting information can be downloaded at: https://www.mdpi.com/article/10.3390/ani16132091/s1, Figure S1: PCoA analysis based on Bray–Curtis distances among microbial communities grouped by system; Table S1: Effect of PE on microbial community on alpha diversity index; Table S2: Effect of ST on microbial community on alpha diversity index; Table S3: Effects of isolate supplementation on growth performance; Table S4: Effect of isolates on microbial community on alpha diversity index of original rumen; Table S5: Effect of isolates on microbial community on alpha diversity index of ex vivo rumen; Table S6: Effect of isolates on microbial community on alpha diversity index of fecal.
