# Supplementary material for: Effects of Oral Administration of Goat Rumen-Derived Bacterial Isolates on In Vitro and In Vivo Rumen Fermentation Characteristics
Source: Animals (Basel). 2026 Jul 6;16(13):2091. doi: 10.3390/ani16132091 (PMC13360081; doi:10.3390/ani16132091)
Supplement: Supplementary file 1 [file animals-16-02091-s001.zip › animals-4389062-supplementary.pdf]

Supplementary Table S1. Effect of PE on microbial community on alpha diversity index

Measure	Control	PE 10^4	PE 10^6	PE 10^8	P value (Kruskal-Wallis H)
Chao1	865.7	879.7	791.4	860.4	0.954
ACE	864.0	877.8	789.9	859.8	0.954
Shannon	5.188	5.155	5.008	5.124	0.557
Simpson	0.983	0.981	0.979	0.98	0.875

Supplementary Table S2. Effect of ST on microbial community on alpha diversity index

Measure	Control	ST 10^4	ST 10^6	ST 10^8	P value (Kruskal-Wallis H)
ACE	755.8	685.7	849.9	860.5	0.764
Chao1	755.7	685.6	853.5	860.6	0.764
Shannon	5.195	5.178	5.153	5.125	0.954
Simpson	0.983	0.984	0.981	0.974	0.287

Supplementary Table S3. Effects of isolate supplementation on growth performance.

**Item**	**Control**	**ST**	**PE**	**SEM**	**P-value**
Initial BW (kg)	23.61	23.60	23.77	6.358	0.831
Final BW (kg)	25.07^{a}	24.87^{ab}	24.73^{b}	6.446	0.050
Total weight gain (kg)	1.453	1.267	0.967	0.251	0.399
Average daily gain (g/d)	69.21	60.32	46.03	11.93	0.399
Daily feed intake (g/d)	595.0	637.3	607.4	105.8	0.068
Feed conversion ratio	12.02	16.49	15.35	2.507	0.284

Supplementary Table S4. Effect of isolates on microbial community on alpha diversity index of original rumen

Measure	Control	ST	PE	P value (Kruskal-Wallis H)
ACE	1999	1877	1897	0.875
Chao1	2001	1877	1898	0.875
Observed	1997	1876	1895	0.925
Shannon	5.472	5.310	5.263	0.301
Simpson	0.984	0.980	0.977	0.051

Supplementary Table S5. Effect of isolates on microbial community on alpha diversity index of ex vivo rumen.

Measure	Control	ST	PE	P value (Kruskal-Wallis H)
ACE	1825	1824	1818	0.837
Chao1	1827	1824	1819	0.837
Observed	1824	1823	1817	0.837
Shannon	5.542	5.505	5.655	0.733
Simpson	0.987	0.985	0.989	0.393

Supplementary Table S6. Effect of isolates on microbial community on alpha diversity index of fecal.

Measure	Control	ST	PE	P value (Kruskal-Wallis H)
ACE	2477	3030	2602	0.288
Chao1	2479	3037	2605	0.288
Observed	2475	3028	2600	0.288
Shannon	6.500	6.483	6.421	0.957
Simpson	0.996	0.995	0.995	0.177

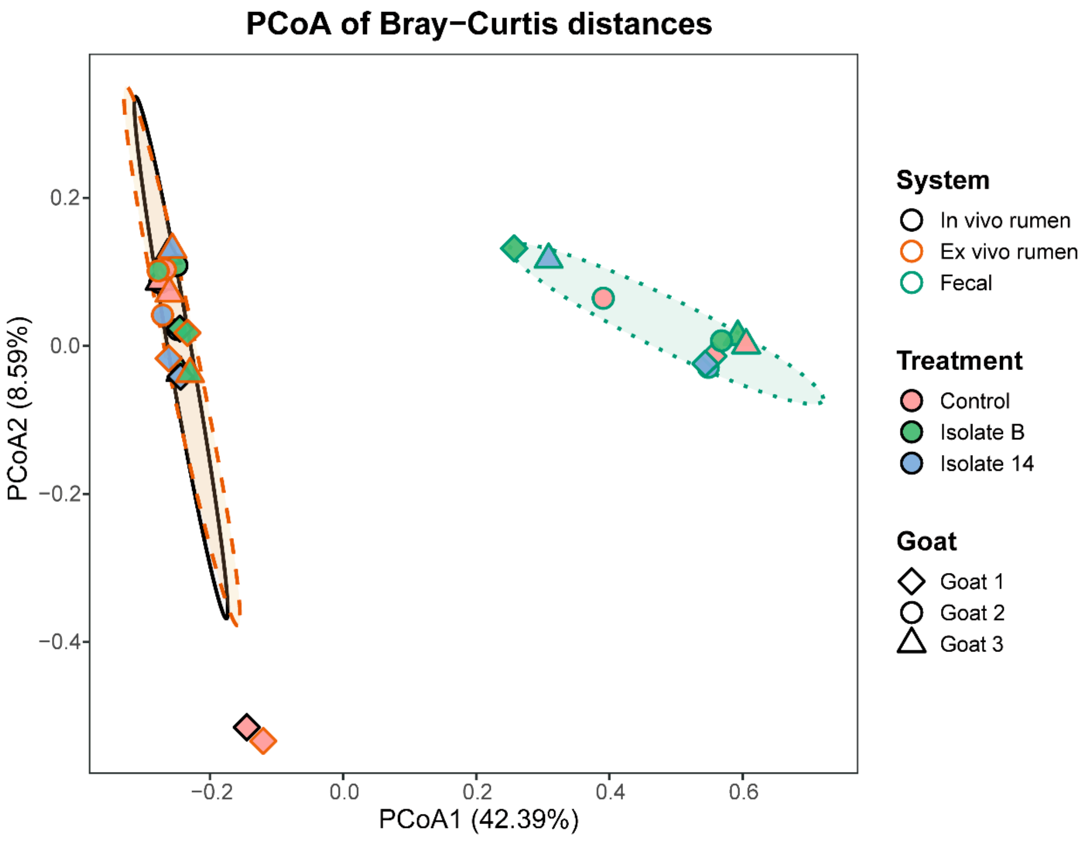

Supplementary Figure S1. PCoA analysis based on Bray–Curtis distances among microbial communities grouped by system
